# Assessing the Prevalence of Male Obesity-Associated Gonadal Dysfunction in Severe Obesity: A Focus on the Impact of Bariatric Surgery and Surgical Approaches

**DOI:** 10.1007/s11695-024-07426-8

**Published:** 2024-07-31

**Authors:** Alessio Bombardieri, Annalisa Bufano, Noemi Fralassi, Cristina Ciuoli, Nicoletta Benenati, Cristina Dalmiglio, Costantino Voglino, Andrea Tirone, Giuseppe Vuolo, Maria Grazia Castagna

**Affiliations:** 1 Department of Medicine, Surgery and Neuroscience, UOC Endocrinology, University of Siena, Siena, Italy; 2 Department of Biochemical, Experimental and Clinical Biomedical Sciences “Mario Serio”, University of Florence, Florence, Italy; 3 Department of General and Specialized Surgery, Unit of Bariatric Surgery, University of Siena, Siena, Italy

**Keywords:** Hypogonadism, Male obesity, Bariatric surgery, Sexual hormones, Testosterone, Weight loss

## Abstract

**Purpose:**

Obesity is an important risk factor for secondary hypogonadism in men. Several studies evaluated the impact of bariatric surgery on gonadal function in men, proving an improvement in testosterone levels, without yet a global consensus on the impact of different surgical approaches. Objectives of the study are: to estimate the prevalence of obesity-associated gonadal dysfunction among men with severe obesity; to evaluate the response to bariatric surgery in terms of resolution of this condition, distinguishing between restrictive and restrictive-malabsorptive surgery.

**Methods:**

We conducted a retrospective evaluation of 413 males with severe obesity (BMI 44.7 ± 8.3 kg/m2). A subgroup of them (61.7%) underwent bariatric surgery. Anthropometric assessment (weight, BMI, waist and hip circumference), metabolic (glyco-lipidic asset and urate) and hormonal (morning gonadotropin and total testosterone) assessments were carried out at baseline and 3–6 months post-surgery.

**Results:**

Using a TT threshold of 2.64 ng/ml, 256 out of 413 (62%) patients were categorized as having biochemical hypogonadism. At multivariate analysis, the only parameter significantly associated with biochemical hypogonadism, was BMI value (*p* = 0.001). At 3–6 months after surgery, during the acute weight loss phase, only 20.1% of patients still had biochemical hypogonadism. At multivariate analysis, which included age, presurgical BMI, pre-surgical TT, surgical approach and %EWL, presurgical TT levels (*p* = 0.0004), %EWL (*p* = 0.04), and mixed restrictive-malabsorptive surgery (*p* = 0.01), were independently associated with the recovery of gonadal function.

**Conclusions:**

The results of this study underscore the potential reversibility of obesity-associated gonadal dysfunction through bariatric surgery, highlighting the importance of considering surgical approach.

**Graphical Abstract:**

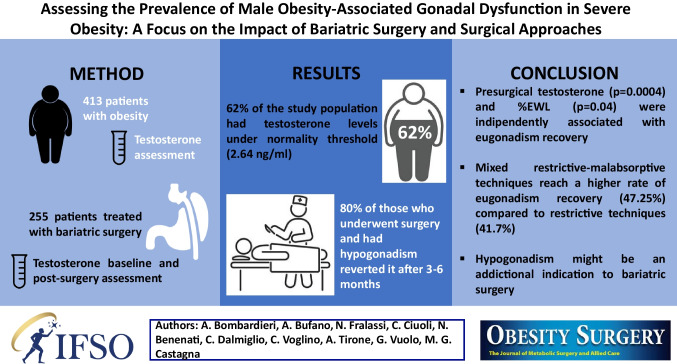

## Introduction

Obesity, a global health epidemic, has emerged as a multifaceted condition with far-reaching implications for various physiological systems [1, 2]. Among the intricate web of health consequences associated with obesity, alterations in endocrine function have gained increasing attention [3–5].

Hypogonadism can be considered a clinical complication of obesity. Obesity results in a 13-fold increase of late-onset secondary hypogonadism [6] and increases of 4–5 kg/m2 in body mass index (BMI) are indeed associated with a reduction in testosterone levels equivalent to about 10 years of aging [7]. Although using different population groups and different total or free testosterone cut-off values, many studies reported a very high prevalence rate of hypogonadism in obese men, that varies from 34% to 68.5% [8, 9]. It is well established that there is a biunivocal relationship between obesity and hypogonadism [8–12]. Morbid obesity represents the major cause of secondary functional hypogonadism [13] and, on the other side, testosterone deficiency promotes adipogenesis and visceral fat deposition and dysfunction [14–16] with consequent chronic inflammation, insulin resistance and decrease of sex hormone binding globulin (SHBG) levels [17, 18].

Because of its functional origin, hypogonadism in obese patients is potentially reversible once treated the underlying etiological cause. It has been proven that a significant weight loss is able to determine an improvement in testosterone (and gonadotropin) levels, often sufficient to shift the patient from hypogonadism to eugonadism status [19–27]. Bariatric surgery currently represents a fundamental therapeutic option to obtain a significant weight reduction in those patients with morbid obesity resistant to lifestyle changes, diet, and pharmacological treatments. In the last decade, several studies have specifically evaluated the impact of bariatric surgery on gonadal function in men, proving an improvement in testosterone levels [20, 28–34]. This improvement was higher with mixed malabsorbitive-restrictive techniques [20, 27], without yet a global consensus on the impact of different surgical approaches.

The aims of this study were as follows: 1) to assess the prevalence of hypogonadism in a substantial cohort of men with obesity; 2) to examine testosterone (TT) levels and their variations at 3–6 months post-surgery in a subgroup of obese male patients who underwent bariatric surgery; 3) to evaluate the impact on recovered hypogonadism of different surgical approaches, including both restrictive and restrictive-malabsorptive methods; 4) to identify potential predictors of gonadal function recovery after surgery.

## Patients and Methods

### Study Population

We conducted a retrospective analysis of 413 male patients with obesity, ranging in age from 18.2 to 69.4 years (mean ± SD, 46.3 ± 12.7), who were referred to the Endocrinology Unit between 2005 and 2020. The inclusion criteria comprised individuals aged over 18 years with a BMI ≥ 30 kg/m2, while exclusion criteria included the use of drugs known to impact the gonadal axis, as well as the presence of pituitary disease or primary hypogonadism. Two hundred and fifty-five/413 (61.7%) patients underwent bariatric surgery at Bariatric Surgery Units. According to the Italian Society of Obesity Surgery (SICOB) and International Federation for the Surgery of obesity and metabolic disorders (IFSO) criteria, indications for bariatric surgery were: BMI ≥ 40 kg/m^2^; BMI ≥ 35 kg/m^2^ with associated co-morbidities; BMI 30–35 kg/m^2^ and type 2 diabetes with poor control despite optimal medical therapy. Different bariatric procedures were performed according to clinical co-morbidities of patients: a restrictive surgical option was used in 55.3% (141/255) of cases [specifically, sleeve gastrectomy in 43.5% (111/255), gastric band in 9.4% (24/255) and placement of an intragastric balloon in 2.4% (6/255)]; a malabsorptive option, i.e. bilio-pancreatic diversion in 3.9% (10/255), and a mixed restrictive-malabsorptive option in 40.8% (104/255) [specifically, One Anastomosis Gastric Bypass (OAGB) in 24.7% (63/255), Roux-en-Y gastric bypass (RYGB) in 16.1% (41/255)].

### Anthropometry

Body weight (kg) and height (m) were measured using a steelyard scale with altimeter (Seca-Intermed, Milan, Italy). BMI was calculated as kg/m2. Waist circumference (WC) was measured in cm, at the midpoint between the upper edge of the iliac crest and the lower costal margin, considered at the level of the middle axillary line. Hip circumference (HC) was measured in cm, on a horizontal plane passing between greater trochanters. Waist-hip ratio (WHR) was calculated as WC/HC. Excess Weight (EW) was calculated using the formula [actual body weight – adjusted body weight]. The adjusted body weight was obtained with the formula: [ideal body weight + 0.4 (actual body weight – ideal body weight). For men, the ideal body weight is 50 kg + 2.3 kg for each inch over 5 feet [35]. At follow-up visit after bariatric surgery, excess weight loss % (EWL%) and total weight loss % (%TWL) were calculated to assess the percentage of weight lost with surgery. To calculate %EWL the formula was

$$\%EWL=[(initial weight-final body weight)/EW]\times 100$$

while, %TWL formula is:

$$\%TWL=[(initial weight-final body weight)/(initial weight)]\times 100.$$

### Hormonal and Biochemical Measurement

A single fasting morning venous blood samples were obtained in all patients to measure luteinizing hormone (LH), follicle-stimulating hormone (FSH), total testosterone (TT), glucose, insulin, HbA1c, total cholesterol, HDL fraction, LDL fraction, triglycerides and uric acid. In surgical patients, the same evaluation (with the exception of FSH and LH) was taken at each follow-up visit. TT was measured by chemiluminescence immunoassays (Beckman Coulter, California, USA), with 0.1 ng/ml analytical sensitivity. Serum LH and FSH were measured by immunochemoluminescence (Beckman Coulter, California, USA), with 0.2 mU/ml analytical sensitivity. The reference intervals validated by internal measurements were: TT 2.64–10.9 ng/ml, LH 0.8–8.0 mU/ml, FSH 0.7–11.0 mU/ml. The other biochemical parameters were performed in hospital laboratory with standardized methods.

### Diagnostic Definitions

Obesity grades were defined as follows: grade I for BMI 30–34.9 kg/m^2^; grade II for BMI 35–39.9 kg/m^2^; grade III for BMI > 40 kg/m^2^, further divided into grade III-A for BMI 40–44.9 kg/m^2^, grade III-B for BMI 45–49.9 kg/m^2^, and grade III-C for BMI > 50 kg/m^2^. WC ≥ 102 cm and WHR > 0.95 were chosen as threshold to define central obesity. Patients with TT < 2.64 ng/ml (or 9.2 nmol/l) were defined as affected by biochemical hypogonadal, according to 2018 American guidelines for male hypogonadism [36]. To define secondary hypogonadism, also LH levels < 9.0 mU/ml were needed. A diagnosis of arterial hypertension was made in the presence of systolic pressure values ≥ 140 mmHg or in the presence of diastolic pressure values ≥ 90 mmHg or in presence of anti-hypertension drugs. In absence of known diagnosis, dyslipidemia was defined as total cholesterol values > 5.17 mmol/L, in presence of LDL cholesterol values > 3.62 mmol/L or in patients with lipid-lowering therapy. In absence of a known diagnosis, we diagnosed diabetes mellitus in the presence of two findings of fasting glucose ≥ 7 mmol/L, in the presence of two findings of glycated hemoglobin ≥ 47.5 mmol/mol or in the presence of a random glucose finding ≥ 11.11 mmol/mol. Metabolic syndrome was diagnosed in the presence of at least three of: systolic blood pressure ≥ 140 mmHg, diastolic blood pressure ≥ 90 mmHg, triglycerides ≥ 1.69 mmol/L, fasting blood glucose ≥ 5.56 mmol/L, diabetes mellitus, HDL-cholesterol < 1.03 mmol/L in men and < 1.29 mmol/L in women, abdominal circumference > 1.02 m in men and > 0.88 m in women [International Diabetes Federation criteria]. Insulin resistance was calculated using the Homeostatic Model Assessment index of Insulin Resistance (HOMA-IR = fasting insulin [U/ml] x fasting glucose [mg/dl] /405) [37]. HOMA-IR values < 2.5 were considered normal.

### Statistical Analysis

Continuous variables with normal distribution were presented as mean ± standard deviation; those with non-normal distribution as median and interval between minimum and maximum values. The pre- and post-treatment comparison of variables with normal distribution was performed with paired t-test, while Wilcoxon test was used for variables with non-normal distribution. Categorical variables were presented as percentages. Proportions were analyzed by Fisher’s exact test. The relationship between two parameters was examined with Pearson’s correlation test.

Parameters associations were also investigated by multiple logistic regression models. In particular we evaluated if age, weight, BMI, EW, WC, HC and HOMA-IR (independent variables, found to be significant at univariate analysis) were indipendently associated with TT levels (dependent variable).

We also evaluated if weight, BMI, EW, WC, HC, metabolic syndrome, insulin, and HOMA-IR (independent variables found to be significant at univariate analysis) were indipendently associated with biochemical hypogonadism (dependent variable). Finally, we evaluated if age, presurgical BMI, pre-surgical TT, surgical approach and %EWL, %TWL and presurgical TT levels as independent variables (those to found to be significant at univariate analysis) were indipendently associated with biochemical eugonadism recovery after surgery (dependent variable).

*P*-value < 0.05 were considered statistically significant. All statistical analyses were conducted using StatView for Windows 5.0.1 (SAS Institute) and SPSS for Windows 15.0 (IBM).

## Results

### Demographic, Anthropometric, Biochemical and Clinical Features of the Study Population at Baseline (*n* = 413)

Table 1 reports demographic, anthropometric, biochemical and clinical features of the study population at baseline. Mean BMI was 44.7 ± 8.3 kg/m^2^ (range 30.2–73.8 kg/m^2^, median 43.9 kg/m^2^), with 9.7% of patients classified as grade I obesity, 22.3% as grade II and 68.0% as grade III (23.0% III-A, 21.3% III-B, 23.7% III-C respectively). Mean EW at baseline was 61.8 ± 25.3 kg (range 17.5–159.0 kg, median 59.3 kg). Diabetes mellitus was found in 43.6% of the population, arterial hypertension in 61.7%, dyslipidemia in 49.4% and metabolic syndrome in 74.6%.

**Table 1:** Demographic, anthropometric and biochemical characteristics of the population at baseline (*n* = 413)

Age (years) mean ± *SD* *(median, range)*	46.3 ± 12.7 (46.6, 18.2–69.4)
Weight (kg) mean ± *SD* *(median, range)*	136.0 ± 27.4 (134.0, 79.6–254.0)
Height (m) mean ± *SD* *(median, range)*	1.74 ± 0.08 (1.73, 1.56–1.95)
BMI (kg/m2) mean ± *SD* *(median, range)*	44.7 ± 8.3 (43.9, 30.2–73.8)
EW (kg) mean ± *SD* *(median, range)*	61.8 ± 25.3 (59.3, 17.5–159.0)
WC (cm) mean ± *SD* *(median, range)*	132.2 ± 13.3 (132.5, 105.0–169.0)
HC (cm) mean ± *SD* *(median, range)*	128.4 ± 13.3 (128.0, 104.0–160.0)
WHR mean ± *SD* *(median, range)*	1.03 ± 0.09 (1.03, 0.81–1.29)
TT (ng/ml) mean ± *SD* *(median, range)*	2.56 ± 1.04 (2.46, 0.55–7.76)
LH (mIU/ml) mean ± *SD* *(median, range)*	3.8 ± 1.9 (3.4, 0.3–11.8)
FSH (mIU/ml) mean ± *SD* *(median, range)*	5.6 ± 3.4 (4.7, 0.7–23.7)
HOMA-IR mean ± *SD* *(median, range)*	8.1 ± 7.9 (5.8, 0.4–68.4)
***Comorbidities***	
Diabetes mellitus (%)	180/413 (43.6%)
Hypertension (%)	255/413 (61.7%)
Dyslipidemia (%)	204/413 (49.4%)
Metabolic syndrome (%)	308/413 (74.6%)

Among biochemical parameters, mean TT was 2.56 ± 1.04 ng/ml (range 0.55–7.76 ng/ml, median 2.46 ng/ml), lower than the threshold value chosen to define hypogonadism. When we related serum TT with obesity categories, we observed no statistically significant differences between grade I and II obesity (2.9 ± 1.44 ng/ml versus 2.85 ± 1.05 ng/ml, *p* = 0.71) while significantly lower values were observed in grade III obesity (2.41 ± 0.99 ng/ml), when compared with grade I (*p* = 0.003) and grade II obesity (*p* = 0.0003).

A linear regression model was employed with TT as the dependent variable and age, weight, BMI, EW, WC, HC and HOMA-IR as independent variables. The analysis revealed a statistically significant negative correlation of TT with weight (*r* = 0.22, *p* < 0.0001), BMI (*r* = 0.25, *p* < 0.0001), EW (*r* = 0.24, *p* < 0.0001), WC (*r* = 0.17, *p* = 0.01), HC (*r* = 0.20, *p* = 0.007), with a trend towards significance for HOMA-IR (*r* = 0.13, *p* = 0.05). Upon conducting multivariate analysis, it was observed that the only parameter independently associated with TT levels was BMI (*p* = 0.001).

### Association of Biochemical Hypogonadism with Anthropometric and Clinical Parameters in Study Population (*n* = 413)

Using a TT threshold of 2.64 ng/ml, 256 out of 413 (62%) patients were categorized as having biochemical hypogonadism. Table 2 presents the demographic, anthropometric, biochemical, and clinical features of patients stratified based on their TT levels. Body weight, BMI, EW, WC, HC, the prevalence of metabolic syndrome, insulin, and HOMA-IR were notably higher in the biochemical hypogonadal group, while age, LH, and TT values were significantly lower. Height, WHR, FSH, and the prevalence of diabetes mellitus, hypertension, and dyslipidemia did not differ between the two groups. At multivariate analysis, the only parameter significantly associated with biochemical hypogonadism, was BMI value (*p* = 0.001).

**Table 2:** Demographic, anthropometric and biochemical characteristics of patients divided upon TT levels (YES TT < 1.82 ng/ml; NO TT > 1.82 ng/ml)(*n* = 413)

	Biochemical hypogonadism		
	YES (*n* = 256/413, 62%)	NO (*n* = 157/413, 38%)	*p*
*Demographic and anthropometric parameters:*			
Age (years) mean ± *SD (median, range)*	45.2 ± 12.8 (45.1, 18.9–69.4)	48.2 ± 12.5 (48.9, 18.2–67.7)	**0.01**
Weight (kg) mean ± *SD (median, range)*	139.8 ± 27.2 (138.0, 79.6–254.0)	129.9 ± 26.7 (128.4, 80.0–242.0)	**0.0003**
Height (m) mean ± *SD (median, range)*	1.74 ± 0.08 (1.73, 1.56–1.95)	1.74 ± 0.08 (1.73, 1.56–1.95)	0.97
BMI (kg/m2) mean ± *SD (median, range)*	45.9 ± 8.4 (45.5, 30.2–73.8)	42.7 ± 7.7 (41.9, 30.5–67.0)	** < 0.0001**
EW (kg) mean ± *SD (median, range)*	65.5 ± 25.3 (64.0, 17.5–159.0)	55.6 ± 24.1 (53.0, 18.0–152.0)	**0.0001**
WC (cm) mean ± *SD (median, range)*	133.8 ± 12.9 (134.0, 105.0–169.0)	128.8 ± 13.4 (126.0, 105.0–166.0)	**0.01**
HC (cm) mean ± *SD (median, range)*	129.9 ± 14.3 (130.0, 104.0–160.0)	125.3 ± 10.4 (126.0, 104.0–155.0)	**0.03**
WHR mean ± *SD (median, range)*	1.03 ± 0.09 (1.03, 0.81–1.29)	1.02 ± 0.08 (1.02, 0.83–1.22)	0.42
*Biochemical parameters:*			
TT (ng/ml) mean ± *SD (median, range)*	1.95 ± 0.51 (2.0, 0.55–2.64)	3.55 ± 0.92 (3.2, 2.66–7.76)	** < 0.001**
LH (mIU/mL) mean ± *SD (median, range)*	3.7 ± 1.9 (3.3, 0.3–8.6)	4.1 ± 1.9 (3.7, 0.3–11.8)	**0.04**
FSH (mIU/mL) mean ± *SD (median, range)*	5.5 ± 3.6 (4.6, 0.7–23.7)	5.7 ± 2.9 (4.9, 1.4–21.9)	0.56
HOMA-IR mean ± *SD (median, range)*	9.02 ± 8.81 (6.4, 0.99–68.44)	6.35 ± 5.64 (4.5, 0.44–27.14)	**0.01**
Insulin (mcU/ml) mean ± *SD (median, range)*	29.6 ± 22.2 (25.0, 4.5–180.0)	22.5 ± 15.2 (17.5, 1.8–80.7)	**0.009**
*Comorbidities*			
Diabetes mellitus (%)	112/256 (43.7%)	68/157 (43.3%)	0.9
Hypertension (%)	150/256 (58.6%)	105/157 (66.9%)	0.09
Dyslipidemia (%)	131/256 (51.2%)	73/157 (46.5%)	0.3
Metabolic syndrome (%)	200/256 (78.1%)	108/157 (68.8%)	**0.03**

**Fig. 1:**
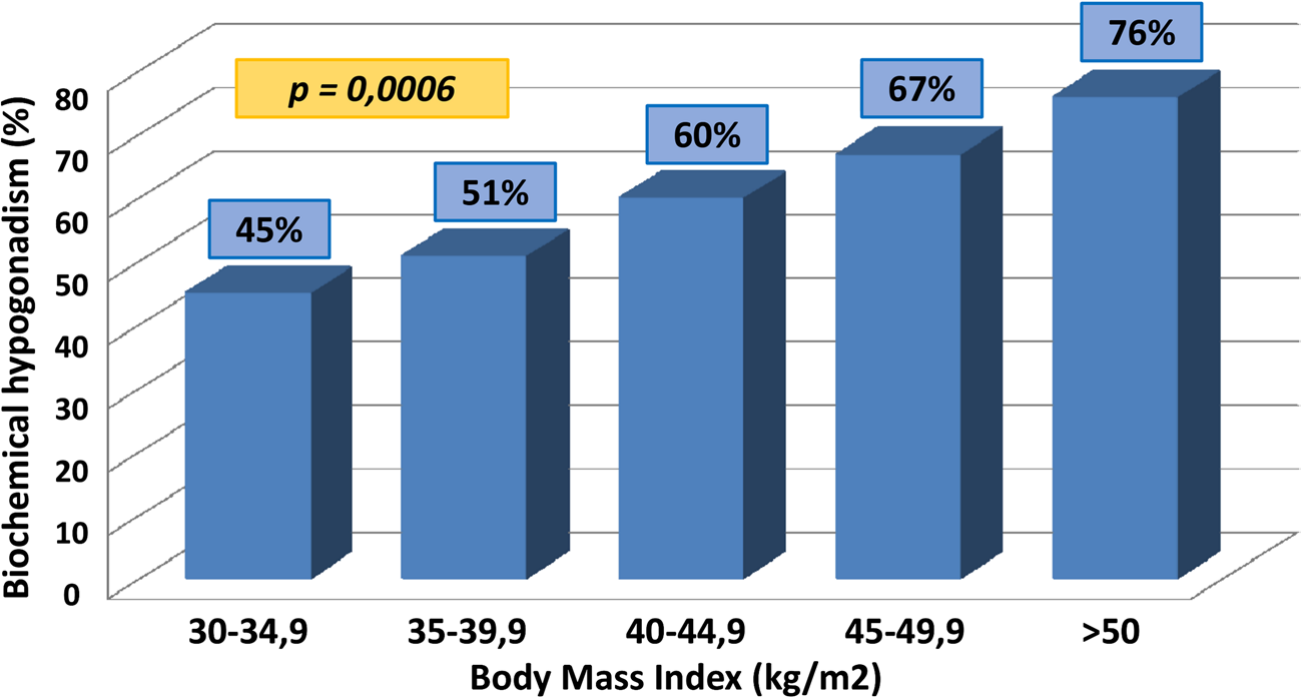
Prevalence of biochemical hypogonadism (TT < 2.64 ng/ml) in obese patients divided by obesity degree

The rate of biochemical hypogonadism prevalence significantly increased as the obesity grade increased (Fig. 1). Biochemical hypogonadism was observed in 45% of subjects with BMI between 30 and 34.9 kg/m^2^, in 51% of those with BMI 35–39.9 kg/m^2^, in 68% of those with BMI > 40 kg/m^2^ (60% with BMI 40–44.9 kg/m^2^, 67% with BMI 45–49.9 kg/m^2^ and 76% with BMI > 50 kg/m^2^, respectively), with statistically significant difference between classes (*p* = 0.0006).

### Analysis of Anthropometric, Biochemical, and Clinical Features in 255 Patients Submitted to Bariatric Surgery

Two hundred and fifty-five out of 413 (61.7%) patients underwent bariatric surgery. Table 3 shows demographic, anthropometric, and biochemical characteristics of the subpopulation of patients who underwent bariatric surgery.

**Table 3:** Demographic, anthropometric and biochemical characteristics of the subpopulation of patients underwent bariatric surgery at baseline (*n* = 255) and post surgery (*n* = 185)

	Baseline *(n* = *255)*	Post-surgery *(n* = *184)*	*p*
Age (years) mean ± *SD (median, range)*	45.5 ± 11.9 (45.7, 18.7–77.7)		/
Height (m) mean ± *SD (median, range)*	1.74 ± 0.07 (1.73, 1.56–1.93)		/
Weight (kg) mean ± *SD (median, range)*	139.2 ± 22.9 (136.9, 92.9–233.2)	105.9 ± 18.3 (103.0, 74.4–203.0)	** < 0.0001**
BMI (kg/m2) mean ± *SD (median, range)*	45.9 ± 7.4 (45.1, 33.7–73.8)	34.8 ± 5.5 (33.8, 23.1–56.2)	** < 0.0001**
EW (kg) mean ± *SD (median, range)*	65.0 ± 21.8 (62.7, 27.0–143.2)	31.4 ± 16.9 (29.0, -5.7–113.0)	** < 0.0001**
WC (cm) mean ± *SD (median, range)*	132.2 ± 13.3 (132.5, 105.0–169.0)	109.8 ± 12.5 (107.5, 79.0–148.0)	** < 0.0001**
HC (cm) mean ± *SD (median, range)*	128.4 ± 13.3 (128.0, 104.0–160.0)	111.5 ± 10.9 (110.0, 87.0–142.0)	** < 0.0001**
WHR mean ± *SD (median, range)*	1.03 ± 0.09 (1.03, 0.81–1.29)	0.99 ± 0.08 (0.98, 0.81–1.23)	** < 0.0001**
HOMA-IR *(median, range)*	8.56 ± 8.05 (6.2, 0.44–68.40)	2.83 ± 3.67 (1.8, 0.30–18.33)	**0.01**
***Comorbidities***			
Diabetes mellitus (%)	83/255 (32.5%)	11/184 (6%)	** < 0.0001**
Hypertension (%)	149/255 (58.4%)	28/184 (15.2%)	** < 0.0001**
Dyslipidemia (%)	126/255 (49,4%)	22/184 (12%)	** < 0.0001**
Metabolic syndrome (%)	198/255 (77,6%)	9/184 (4.9%)	** < 0.0001**

The mean BMI at baseline was 45.9 ± 7.4 kg/m^2^ (range 33.7–73.8, median 45.1), with only 1.9% of patients classified as grade I obesity, 21.2% as grade II, and 76.9% as grade III (25.9% III-A, 23.5% III-B, 27.5% III-C, respectively). The mean excess weight (EW) at baseline was 65.0 ± 21.8 kg (range 27.0–143.2, median 62.7). Diabetes mellitus was found in 32.5% of the population, arterial hypertension in 58.4%, dyslipidemia in 49.5%, and metabolic syndrome in 77.6%.

All patients were re-evaluated 3–6 months after bariatric surgery. The mean post-surgical BMI was 34.8 ± 5.5 kg/m^2^ (range 23.1–56.2 kg/m^2^, median 33.8 kg/m^2^), significantly lower than that observed at baseline (45.9 ± 7.4 kg/m^2^, range 33.7–73.8 kg/m^2^, median 45.1 kg/m^2^; *p* < 0.0001). Almost 20% of patients had a BMI < 30 kg/m^2^, no longer indicative of obesity; 38.6% had grade I obesity, 23.7% had grade II, and 17.8% had grade III (13.1% III-A, 3.8% III-B, 0.9% III-C, respectively). The mean post-surgical EW was 31.4 ± 16.9 kg (range -5.7–113.0 kg, median 29.0 kg), significantly lower than that observed at baseline (*p* < 0.0001). At 3–6 months after surgery, the rate of patients with diabetes mellitus, arterial hypertension, dyslipidemia, and metabolic syndrome was significantly lower than that observed at baseline (*p* < 0.0001). Three to six months after surgery, remission from diabetes mellitus occurred in 80% of patients (44/55), from hypertension in 73% of patients (76/104), and from dyslipidemia and metabolic syndrome in 75.3% (67/89) and 93.9% (140/149) of patients, respectively.

### Pre-and Post-Surgical Testosterone Levels in Patients Undergoing Bariatric Surgery

**Fig. 2:**
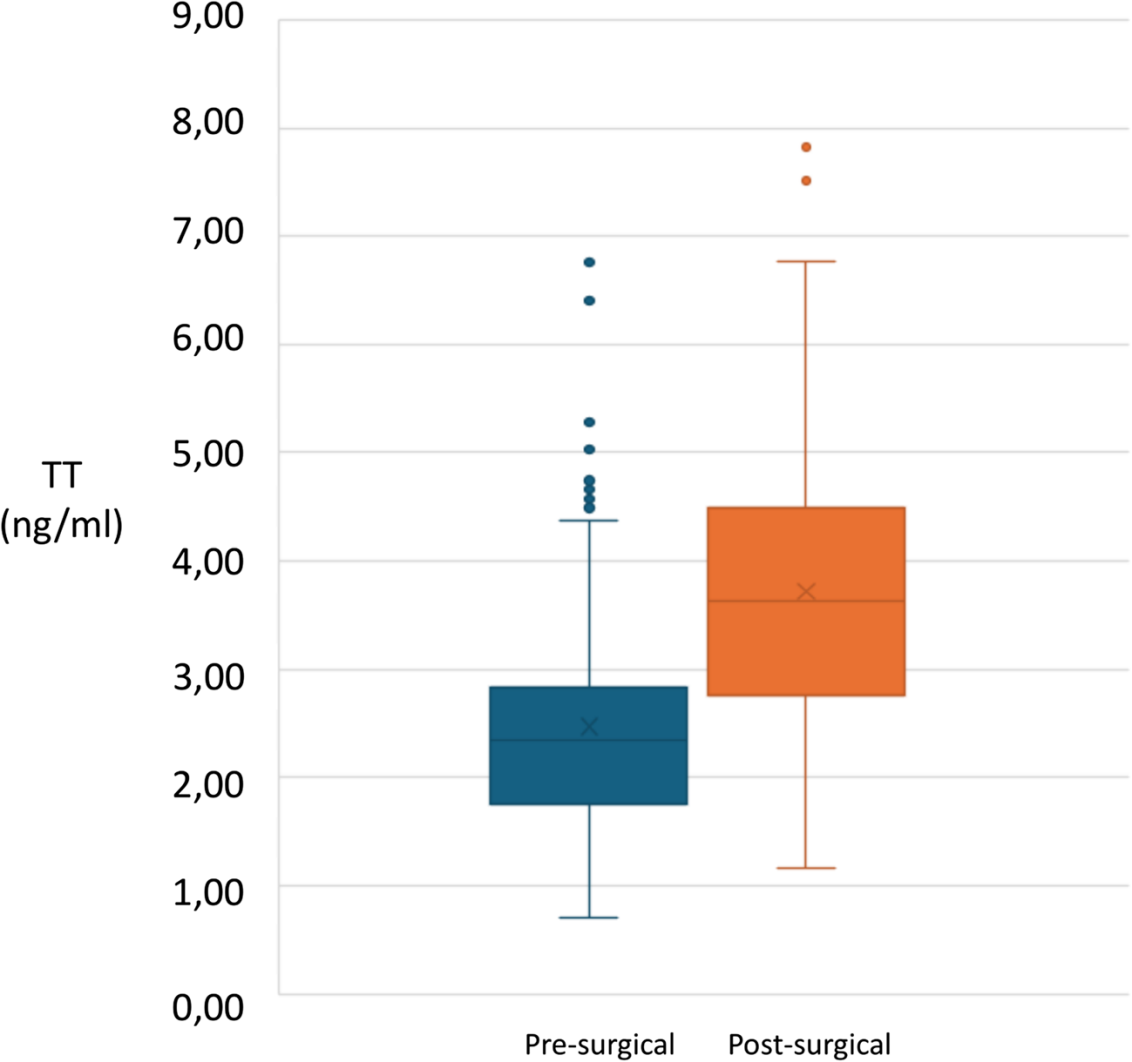
Pre-surgical and post-surgical TT (ng/ml) distribution in the population undergone bariatric surgery (*n* = 184). (*p* = 0.002)

Pre-surgical TT, available in 255 patients, was 2.51 ± 0.99 ng/ml (range 0.70–6.78 ng/ml, median 2.40 ng/ml), still lower than the hypogonadism threshold. Post-surgical TT was available in 184/255 (72%) patients, and the mean TT value was 3.72 ± 1.26 ng/ml (range 1.16–7.87 ng/ml, median 3.63 ng/ml), significantly higher than that observed, in the same subject, before bariatric surgery (*p* < 0.0001) (Fig. 2). At 3–6 months after surgery, during the acute weight loss phase, only 37/184 patients (20.1%) still had low TT, while 147/184 (79.9%) showed normal TT levels. The rate of post-surgical biochemical hypogonadism was significantly lower than that observed, in the same cohort of patients before bariatric surgery (69.5% versus 20.1%, *p* = 0.0028). Specifically, 74.2% (95/128) of biochemically hypogonadic patients were classified as eugonadic after bariatric surgery.

### Predictors of Testosterone Levels Normalization after Bariatric Surgery (*n* = 128)

As shown in Table 4, at the univariate analysis, basal TT levels were significantly higher in patients who were biochemically eugonadic after bariatric surgery (2.03 ± 0.4 ng/ml) compared to those with persistent biochemical hypogonadism (1.69 ± 0.55 ng/ml; *p* = 0.02). %EWL was significantly higher in patients who were eugonadic after bariatric surgery compared to those with persistent biochemical hypogonadism (57.04 ± 15.04% vs 47.63 ± 15.4%, *p* = 0.005). Similarly, %TWL was significantly higher in those who reverted (25.32 ± 7.2% vs 21.47 ± 8.2% in those who persisted hypogonadic; *p* = 0.027).

**Table 4:** Predictors of testosterone levels normalization after bariatric surgery

*Pre-surgical data*	*Persistent hypogonadism* (*n* = 33/128, 25.8%)	*Eugonadism recovery* (*n* = 95/128, 74.2%)	*p*
Age (years) mean ± *SD*	45.0 ± 9.6	42.7 ± 12.1	0.2
Presurgical weight (kg) mean ± *SD*	138.0 ± 26.54	139.2 ± 22.03	0.5
BMI (kg/m2) mean ± *SD*	46.2 ± 8.5	45.7 ± 7.1	0.9
EW (kg) mean ± *SD*	65.2 ± 24.6	64.6 ± 21.0	0.8
WC (cm) mean ± *SD*	130.6 ± 14.3	133.3 ± 12.4	0.2
HC (cm) mean ± *SD*	126.9 ± 12.98	130.9 ± 145.2	0.2
WHR mean ± *SD*	1.02 ± 0.09	1.02 ± 0.09	0.9
TT (ng/ml) mean ± *SD*	1.69 ± 0.55	2.03 ± 0.4	**0.002**
LH (mIU/mL) mean ± *SD*	3.2 ± 1.6	3.83 ± 1.9	0.1
FSH (mIU/mL) mean ± *SD*	4.6 ± 2.63	5.03 ± 2.9	0.5
HOMA-IR mean ± *SD*	9.5 ± 8	7.98 ± 6.1	0.5
***Post-surgical data***			
Remission from metabolic syndrome (%)	22/26 (84.6%)	66/69 (95.6%)	**0.06**
EWL (%)	47.63 ± 15.4	57.04 ± 15.4	**0.005**
TWL (%)	21.47 ± 8.2	25.32 ± 7.2	**0.027**

We also analyzed the recovery rate of eugonadism based on three different surgical approaches: mixed restrictive-malabsorptive surgery (specifically RYGB and OAGB), restrictive surgery (sleeve gastrectomy), and minimally invasive restrictive surgery (gastric band). Intra-gastric balloon and malabsorptive bilio-pancreatic diversion were not included in the analysis due to the small sample size. The higher rate of eugonadism recovery was observed in patients treated with mixed restrictive-malabsorptive surgery (47.25% versus 41.7% with restrictive techniques and 11% with gastric band, *p* = 0.02).

At multivariate analysis, which included age, presurgical BMI, pre-surgical TT, surgical approach, %EWL and presurgical TT levels (*p* = 0.0004), %EWL (*p* = 0.04), and mixed restrictive-malabsorptive surgery (*p* = 0.01), were independently associated with the recovery of gonadal function. Performing the same analysis with %TWL instead of %EWL, we did not find any significant correlation between %TWL and recovery rate of eugonadism (*p* = 0.09).

## Discussion

Obesity, defined by an excess accumulation of body fat, has witnessed an alarming rise in prevalence worldwide. Beyond its well-established links with cardiovascular diseases, diabetes, and musculoskeletal issues, obesity’s impact on reproductive health has become an area of intense investigation [5, 38, 39]. Hypogonadism, marked by reduced testosterone levels, stands out as a noteworthy consequence of obesity, affecting both males and females [13, 40–44]. Biochemical hypogonadism, defined by a testosterone (TT) threshold of 2.64 ng/ml, was observed in a substantial percentage (62%) of our study population. This aligns with existing literature indicating a link between obesity and hypogonadism [9]. The association of lower TT values with increasing obesity grades, as demonstrated in the study, corroborates findings in the literature [45]. Moreover, we demonstrated how BMI was the only independent parameter associated with low TT levels and obesity-related biochemical hypogonadism rate increased linearly with increasing BMI class (45% for BMI 30–34.9 kg/m^2^, 51% for BMI 35–39.9 kg/m^2^ and 68% for BMI > 40 kg/m^2^).

The post-surgical outcomes highlight the efficacy of bariatric surgery in inducing significant weight loss and improving metabolic health. The remarkable reduction in BMI from 45.9 to 34.8 kg/m^2^, along with a substantial decrease in excess weight, signifies the success of the surgical intervention in achieving meaningful weight loss. The re-evaluation at 3–6 months post-surgery unveils a noteworthy shift in the distribution of obesity grades, with a considerable proportion of patients transitioning to lower obesity categories. This transformation is particularly evident in the decline of patients with grade III obesity, indicating a positive impact on overall health status.

The presented results offer valuable insights into the impact of bariatric surgery on testosterone levels in a sub-cohort of 255 patients, providing a nuanced perspective on the intersection between obesity, surgery, and hormonal regulation. After bariatric surgery, a significant elevation in testosterone levels was observed, with a mean of 3.72 ng/ml. This substantial increase was statistically significant and underscores the positive impact of weight loss through surgical intervention on hormonal regulation. The noteworthy improvement in testosterone levels post-surgery is consistent with the broader understanding that weight loss can positively influence endocrine function [46–48].

The reduction in the rate of post-surgical biochemical hypogonadism, from 69.5% pre-surgery to 20.1% post-surgery, is a key finding. This shift underscores the effectiveness of bariatric surgery in improving biochemical hypogonadism in a significant proportion of patients. The fact that only 20.1% of patients maintained low testosterone levels during the acute weight loss phase, while the majority achieved normal levels, points towards a robust and rapid response to the surgical intervention.

These results are in line with existing literature that highlights the beneficial impact of weight loss, particularly through bariatric surgery, on testosterone levels [19, 21]. However, the majority of published studies include small cohorts of mostly obese patients, primarily treated with a single surgical procedure, predominantly represented by gastric bypass [49, 50]. In contrast, our study population comprises nearly 200 patients with severe obesity treated with differente surgical approach, allowing us to gather information also on the effectiveness of various bariatric surgery procedures in restoring eugonadism. While a global consensus on the impact of various surgical approaches has not yet been reached, some authors argue that malabsorptive surgery [28, 31, 43, 44] can lead to greater clinical and metabolic improvements compared to non-malabsorptive restrictive surgical techniques [29, 30]. On the other hand, some researchers assert that different techniques yield similar results [8]. It is important to note that studies evaluating surgical approaches have mostly been conducted on small sample sizes [8]. Our data suggest that patients undergoing mixed restrictive-malabsorptive surgery (specifically RYGB and OAGB) experienced a higher rate of eugonadism recovery (47.25%) compared to those with restrictive techniques (41.7%) and gastric band (11%). This finding aligns the efficacy of mixed surgical approaches in achieving comprehensive health outcomes post-bariatric surgery [51].

Age may contribute to hormonal changes, and BMI reflects the severity of obesity, both of which could impact gonadal function. However, their lack of significance in the multivariate analysis suggests that their influence on gonadal recovery may be mediated or overshadowed by other factors. The significance of presurgical TT levels (*p* = 0.0004) underscores its role in predicting the recovery of gonadal function. It suggests that individuals with specific presurgical TT levels may exhibit a different pattern of gonadal recovery post-bariatric surgery, emphasizing the importance of considering the hormonal baseline. The type of surgical approach employed emerges as a significant factor independently associated with gonadal function recovery (*p* = 0.01). Specifically, mixed restrictive-malabsorptive surgery demonstrates a noteworthy impact. The percentage of excess and total weight loss (%EWL) is a key metric reflecting the success of bariatric interventions. Its inclusion in the analysis highlights its independent association with gonadal function recovery (*p* = 0.04). This suggests that the degree of weight loss, beyond other factors, plays a distinct role in influencing hormonal outcomes, reinforcing the holistic impact of weight reduction on overall health. Understanding the independent associations revealed by the multivariate analysis has important clinical implications. Surgeons and healthcare professionals can use this information to tailor interventions based on patient characteristics and expected outcomes. The identified variables, including presurgical TT levels, surgical approach and %EWL, offer valuable guidance for optimizing patient outcomes in the context of hormonal improvements.

Some of our findings might be integrated in daily clinical practice. A severely reduced testosteronemia at baseline could be a further indication to choose a mixed restrictive-malabsorptive approach over a restrictive one; on the other side. We can speculate that a weight loss achieved by medication and/or diet, which is at the moment inferior to the surgical one, might not be enough to revert biochemical hypogonadism and this patients should be screened and eventually treated.

While these findings offer valuable insights, it is essential to acknowledge potential limitations, such as the retrospective nature of the study. Additionally, we defined biochemical secondary hypogonadism by considering serum total testosterone (TT) and gonadotropin levels, without evaluating free testosterone levels. This likely led to overdiagnosis, as obesity generally inhibits the insulin-mediated hepatic release of SHBG, resulting in its reduction [52–54]. Moreover, we did not use symptom questionnaires addressing sexual and general symptoms associated with testosterone deficiency, and seminal fluid parameters were not examined.

Despite these limitations, this study is, to our knowledge, the first to assess the post-bariatric gonadal state in a large cohort of patients with severe obesity during the early follow-up. It specifically focuses on the rapid weight loss phase, rather than waiting for 6–12 months, demonstrating that the beneficial effects of surgery are early and concurrent with weight loss.

In conclusion, these results contribute valuable insights into the nuanced relationship between bariatric surgery, weight loss, and gonadal function recovery. The findings align with existing literature, providing a foundation for further research and clinical applications in optimizing the holistic benefits of bariatric interventions. Additionally, the study may benefit from exploring additional variables or interactions to enhance the depth of understanding.

## Data Availability

The data that support the findings of this study are not publicly available due to their containing information that could compromise the privacy of research participants but are available from corresponding author MG.C. upon reasonable request.
